# National indicators of health literacy: ability to understand health information and to engage actively with healthcare providers - a population-based survey among Danish adults

**DOI:** 10.1186/1471-2458-14-1095

**Published:** 2014-10-22

**Authors:** Anne Bo, Karina Friis, Richard H Osborne, Helle Terkildsen Maindal

**Affiliations:** Department of Public Health, Section for Health Promotion and Health Services, Aarhus University, Aarhus, Denmark; Public Health and Quality Improvement, Central Denmark Region, Aarhus, Denmark; Public Health Innovation, Population Health Strategic Research Centre, School of Health and Social Development, Deakin University, Melbourne, Australia

**Keywords:** Health literacy, Health competence, Patient-centred care, Patient participation, Doctor-patient relations, Health information, Socioeconomic factors, Inequality in health, Population survey, Health literacy questionnaire (HLQ)

## Abstract

**Background:**

Health literacy is a multidimensional concept covering a range of cognitive and social skills necessary for participation in health care. Knowledge of health literacy levels in general populations and how health literacy levels impacts on social health inequity is lacking. The primary aim of this study was to perform a population-based assessment of dimensions of health literacy related to understanding health information and to engaging with healthcare providers. Secondly, the aim was to examine associations between socio-economic characteristics with these dimensions of health literacy.

**Methods:**

A population-based survey was conducted between January and April 2013 in the Central Denmark Region. Postal invitations were sent to a random sample of 46,354 individuals >25 years of age. Two health literacy dimensions were selected from the Health Literacy Questionnaire (HLQ™): i) Understanding health information well enough to know what to do (5 items), and ii) Ability to actively engage with health care providers (5 items). Response options ranged from 1 (very difficult) to 4 (very easy). We investigated the level of perceived difficulty of each task, and the associations between the two dimensions and socio-economic characteristics.

**Results:**

A total of 29,473 (63.6%) responded to the survey. Between 8.8%, 95% CI: 8.4-9.2 and 20.2%, 95% CI: 19.6-20.8 of the general population perceived the health literacy tasks as difficult or very difficult at the individual item level. On the scale level, the mean rating for i) understanding health information was 3.10, 95% CI: 3.09-3.10, and 3.07, 95% CI: 3.07-3.08 for ii) engagement with health care providers. Low levels of the two dimensions were associated with low income, low education level, living alone, and to non-Danish ethnicity. Associations with sex and age differed by the specific health literacy dimension.

**Conclusion:**

Estimates on two key dimensions of health literacy in a general population are now available. A substantial proportion of the Danish population perceives difficulties related to understanding health information and engaging with healthcare providers. The study supports previous findings of a socio-economic gradient in health literacy. New insight is provided on the feasibility of measuring health literacy which is of importance for optimising health systems.

## Background

With the growing complexity of modern health care, the population is confronted with increasing demands to understand and utilize health information. Together with the increasing proportion of people living with chronic conditions, competencies for proactive self-management of health and participation in collaborative care have become key public health agendas [1, 2]. The ability to take active part in shared decision making with healthcare providers is important for adherence to treatment, self-management of chronic diseases [2–4], and it is rooted in ethical considerations for patient autonomy [5, 6]. However, the World Health Organization recently described a ‘health decision making paradox’ where the increased demands on the individual to make choices for health, are not accompanied by appropriate information and support to enable this to happen [7].

In this context, health literacy has received increased attention as it addresses citizens’ motivation and ability to gain access to, understand and use information in ways which promote and maintain good health [8]. The concept has evolved from a focus on functional skills such as reading and understanding health information to a focus in newer definitions on higher order competencies such as critical appraisal of health information and applying it in everyday life and when interacting with the health care system [9, 10]. The level of skills and competencies required by individuals varies with the contextual demands such as the complexity of the health care system, access to health information or patient education, communication skills of health professionals and their time to convey messages and offer patient support, and the availability of social mobilization or advocacy [11–13]. Also, individuals with more complex conditions face higher management demands.

Health literacy research has its roots in investigations of general adult literacy and health for example the International Adult Literacy Survey and the Adult Literacy and Life Skills Survey [14], and has developed to specifically include health literacy for example in a large health literacy survey from the U.S. using the Health Activities Literacy Scale [15]. Studies have found associations between functional health literacy skills and increased levels of hospitalization, poor adherence to treatment, poorer self-care management of chronic disease, lower participation in screening programs, and higher rates of morbidity and mortality [16, 17].

Expanded conceptual frameworks and comprehensive measurement tools for health literacy have recently been developed [9, 11, 18–20]. These deal with both functional skills and higher order health literacy competencies and they use self-report of health literacy related behaviour or self-reported difficulty of health literacy related tasks. In 2012 the European Health Literacy Consortium performed a health literacy survey (HLS-EU) using a self-report measurement tool in a random sample of approximately 1000 citizens in eight European countries [21]. This study suggested that almost every second citizen has some degree of limited health literacy, as defined by the authors, and limited health literacy was associated with both socio-economic factors and health status [21]. Osborne *et al.* developed a health literacy conceptual model using consultation approaches grounded in the daily lives of citizens, practitioners and policymakers. From this the multi-dimensional Health Literacy Questionnaire (HLQ™) was developed to cover nine separate dimensions of health literacy from functional levels such as understanding and appraising health information to higher order competencies such as interacting with health care providers and actively managing own health [11]. In our study, health literacy is understood in line with these broad conceptualisations as a multidimensional concept encompassing individual skills and competencies at various levels and these have to be understood and interpreted relatively to the contextual demands.

A socio-economic gradient in health literacy across several socio-economic indicators has been observed in previous studies using both functional tests and self-reported measurement tools, with low health literacy levels observed in population groups with lower levels of education and income, and in ethnic minority groups [21–23]. The increasing demands on individuals to take responsibility for their own health seems to inadvertently increase social inequalities in health as it favours those with high health literacy and education levels [24].

Most research on health literacy is based on small samples, focusses on functional health literacy or specific patient groups [7, 25, 26]. No published studies have focused on health literacy in Denmark or other Nordic countries. There is a need for an expanded understanding of health literacy at the population level to guide health services in their responds to the needs of citizens.

The aim of this study was first to describe the level of two important dimensions of health literacy in the Danish population; 1) the ability to understand health information well enough to know what to do, and 2) the ability to actively engage with healthcare providers, and secondly to examine pertinent relationships between socio-economic characteristics with these dimensions of health literacy.

## Methods

### Study design and data collection

A population-based cross sectional study was conducted. Denmark has about 5.5 million inhabitants and is administratively divided into five regions. A sample of 46,354 individuals above 25 years of age was drawn randomly from the Danish Civil Registration System among citizens in the Central Denmark Region, where approximately 22% of the Danish population resides. A total of 29,473 respondents completed and returned the questionnaire giving a response rate of 63.6%.

Data were collected from January to April 2013 through the Central Denmark Region’s health survey that was a part of the Danish National Health Survey [27] in 2013. The national survey was sent out to five mutually exclusive random sub-samples in different regions of Denmark. The National Health Survey contains 54 core items on: health, health behaviour, disease, and socio-economic characteristics. In addition, each region can add separate questions. In 2013, ten questions on two dimensions of health literacy were added in The Central Denmark Region’s health survey. Participants were recruited by mail with an introduction letter describing the purpose and content of the survey. The wording and lay out of the material was fairly simple but not specifically targeted low literate groups and only provided in Danish. The letter emphasized that participation was voluntary and that data would be treated confidentially. The survey applied a self-administered paper or web-based questionnaire, with a reminder procedure comprising three postal reminders.

#### Calculation of population weights

Using the unique personal identification number given to all citizens from the Civil Registration System, both respondents and non-respondents were linked to Danish national registers. We therefore had the opportunity to estimate weights to account for differences in selection probabilities and for differences in response rates for different sub-groups using a model-based calibration approach [28]. Data were weighted to represent the population in the Central Denmark Region. The weights were based on register information on sex, age, municipality of residence, highest completed educational level, income, marital status, country of birth, visit to the general practitioner, hospitalization, occupational status, owner/tenant status, and protection from inquiries during statistical and scientific surveys for all individuals [27].

### Assessment of variables

#### Dependent variables

The health literacy questions were selected from the HLQ™ [11]. The HLQ™ was developed using a validity-driven approach including in depth grounded consultations (workshops and interviews), psychometric analyses, and cognitive interviews. It was initially calibrated among 634 individuals and then confirmed in a replication sample of 405 individuals [11]. The HLQ™ contains 44 questions across nine independent scales. Given that we were only able to include about 10 items, we selected two scales that strongly reflected core and distinct competencies for participation in the health care process: Understand health information well enough to know what to do (‘Understanding’, 5 items), and: ability to actively engage with healthcare providers (‘Engagement’, 5 items). The ten items covered a range of simple or easy through to more challenging heath literacy-related tasks. For each item, participants indicated their perceived difficulty with the response options; 1= very difficult, 2= difficult, 3= easy, and 4= very easy. Scale scores were used as dependent variables and were calculated for each individual as the mean of item scores for the five items. If responses to more than two items in a scale were missing, the scale score was regarded as missing, if one or two items were missing, the mean of the available items was used as the scale score. We did not set a threshold for low or inadequate levels of health literacy, as this would be an arbitrary choice. Table 1 shows the questions and interpretation of scale scores. The questions were presented in a random order in the questionnaire. The translation and adaption of the HLQ™ followed a standardised procedure [29] led by Maindal, Osborne and colleagues to ensure cross-cultural validity. The translation went through five steps including a forward-backward translation, guided by explicit item intent guidance, expert panel discussion, pre-test, and a cognitive testing (manuscript in preparation).

**Table 1:** **Health literacy items and interpretation of scale scores for the two health literacy scales, Central Denmark Region 2013**

Understand health information well enough to know what to do (‘Understanding’)	
1a	*Confidently fill medical forms in the correct way*
2a	*Accurately follow the instructions from…* ^1^
3a	*Read and understand written health information*
4a	*Read and understand all the information on medication labels*
5a	*Understand what healthcare providers are asking you to do*
	**High:** Individuals with high scores are able to understand all written information (including numerical information) in relation to their health and able to write appropriately on forms where required
	**Low:** Individuals with low scores have problems understanding any written health information or instructions about treatments or medications. Unable to read or write well enough to complete medical forms
**Ability to actively engage with healthcare providers (‘Engagement’)**	
1b	*Make sure that healthcare providers understand your problems properly*
2b	*Feel able to discuss your health concerns with a healthcare provider*
3b	*Have good discussions about your health with doctors*
4b	*Discuss things with healthcare providers until you understand all you need to*
5b	*Ask healthcare providers questions to get the health information…* ^1^
	**High:** Individuals with high scores are proactive about their health and feel in control in relationships with healthcare providers. Are able to seek advice from additional healthcare providers when necessary. They keep going until they get what they want. Empowered
	**Low:** Individuals with low scores are passive in their approach to healthcare, inactive i.e., they do not proactively seek or clarify information and advice and/or service options. They accept information without question. Unable to ask questions to get information or to clarify what they do not understand. They accept what is offered without seeking to ensure that it meets their needs. Feel unable to share concerns. The do not have a sense of agency in interactions with providers

^1^Some HLQ™ items are truncated. HLQ is protected by copyright and cannot be used without permission of the authors. Full copy of the items is available at hlq@deakin.edu.au.

#### Demographic and socio-economic characteristics

Demographic and socio-economic factors included: sex, age, ethnicity, education level, personal annual gross income, and cohabitation. Information on sex, age and whether an individual was an immigrant or Danish were collected from national registers to avoid missing data. All other data were self-reported. Immigrants and Danes were identified in registers using Statistics Denmark’s definition [30]: Immigrants are foreign-born with neither parent being both Danish citizens and born in Denmark. Descendants are born in Denmark with neither parent both being Danish citizen and born in Denmark. Danes have at least one parent born in Denmark with Danish citizenship. In this study, immigrants and descendants were grouped in the category “immigrants”, and we denoted the variable ‘Origin’. Mother tongue was grouped as Danish or other language. Using the education nomenclature (ISCED) from Statistics Denmark, educational level was grouped into three categories; low (1–10 years), medium (11–14 years of education), and high (>15 years). Respondents were asked to indicate their approximate gross annual income by marking one of eight income intervals that were chosen based on the general income distribution in the Danish population. For the analysis we grouped the intervals two by two to form the intervals; 0–149,000 DKK, 150,000-374,000 DKK, 375,000-699,000 DKK, >700,000 DKK (7.46 DKK equals 1 €). Cohabitation included whether an individual lives alone or lives with others (adults and/or children). Finally, we collected information on whether a participant had seen a general practitioner within the last year or not. We assumed that individuals who have not seen a general practitioner in the last year might answer questions about engagement differently, as they had to relate to experiences more than a year before. Further, the number of visits to the general practitioner is known to be associated with socio-economic factors [31]; hence the variable was considered a potential confounder.

### Statistical analysis

As the Danish version of the HLQ™ was used for the first time in a large population in the health survey we investigated basic psychometric properties. For each item, proportions of non-response and population-weighted proportions of respondents in each response category were calculated. The correlation between the two scales was investigated using Pearson’s correlation coefficient, and we explored internal consistency using Cronbach’s alpha.

The level of perceived difficulty (difficulty level) of each item was calculated as the population-weighted proportion of respondents who perceived the items as difficult or very difficult with 95% confidence intervals (CI). We calculated the difficulty level of all items for the whole sample and for socio-economic subgroups. Population-weighted multivariate linear regression models were used to assess the associations between socio-economic characteristics and the two dimensions of health literacy on a scale level. Statistical analyses were performed using STATA 13 (StataCorp LP. College Station, TX, USA.).

### Ethics

According to Danish law, approval by the Ethics Committee and written informed consent is not required in questionnaire-based and register-based studies [32]. The provision of information about the survey and its purpose, as well as the voluntary completion and return of the survey by participants constituted implied consent. The study was approved by the Danish Data Protection Agency (j.no: 2007-58-0010) and was undertaken in accordance with the Helsinki Declaration.

## Results

### Psychometric properties

Non-response in the ten health literacy items was low and evenly distributed (between 5.9% and 7.3%) (Table 2), suggesting that items were understood and had acceptable content. For all items, all response options were endorsed by some respondents although there were fewer in the extreme ‘very difficult’ category and many in the “easy” category (Table 2). The population-weighted mean rating for ‘Understanding’ was 3.10, 95% CI: 3.09-3.10, which was marginally higher than for ‘Engagement’ 3.07, 95% CI: 3.07-3.08. Median scores on both scales were three (i.e., perceived as easy). The scales correlated positively with Pearson’s coefficient= 0.76. The Cronbach’s alpha coefficient indicated high internal consistency of both scales; ‘Understanding’ α= 0.87 and ‘Engagement’ α= 0.91.

**Table 2:** **Item missing, population-weighted response distribution and difficulty level of items**

	Item missing	Population-weighted ^1^ proportion in each response category				Population-weighted proportion of population reporting difficult or very difficult across items
Items ^2^		Very difficult	Difficult	Easy	Very easy	
	%	%	%	%	%	% (95% CI)
**Understanding** ^**2**^						
**1a**	6.9	2.6	13.4	57.2	26.7	16.0 (15.5 - 16.6)
**2a**	7.3	1.4	13.1	61.3	24.2	14.5 (14.0 - 15.0)
**3a**	6.7	2.0	10.8	58.3	28.9	12.8 (12.4 - 13.3)
**4a**	6.4	3.2	17.0	56.0	23.8	20.2 (19.6 - 20.8)
**5a**	7.0	1.0	7.8	64.7	26.6	8.8 (8.4 - 9.2)
**Engagement** ^**2**^						
**1b**	6.8	2.5	15.8	57.8	24.0	18.3 (17.7 - 18.8)
**2b**	6.5	1.7	12.8	57.4	28.2	14.5 (14.0 – 15.0)
**3b**	5.9	2.2	14.4	56.2	27.3	16.6 (16.1 - 17.1)
**4b**	7.3	2.0	16.3	56.9	24.8	18.3 (17.8 - 18.9)
**5b**	7.0	1.6	13.7	59.2	25.5	15.3 (14.8 - 15.8)

n= 29,473, Central Denmark Region 2013.

^1^The sample is weighted based on register data to represent the population of the Central Denmark Region 2013.

^2^See Table 1 for description of items and scales.

### Sample characteristics

Characteristics of the respondents are presented in Table 3. There were slightly more women than men, 52.5% versus 47.5%, most were aged 45–64 years (mean age: 54.8, SD 15.3), and about one in 20 were born outside Denmark or had a non-Danish mother tongue. When comparing the distribution of respondents in the sample with the population-weighted distribution also presented in Table 3, it appears that some groups are less represented among the respondents than in the general population, for example the youngest age groups, where the percentage is 27.5% in the sample and 36.6% in the weighted population. Missing data on socio-economic characteristics was low; 5.3% for income and zero for register-based data (sex, age and origin).

**Table 3:** **Participant characteristics and population-weighted difficulty level by socio-economic characteristics, Central Denmark Region 2013**

	Distribution in sample			Population-weighted ^1^ proportion of population reporting difficult or very difficult across items ^2^									
				Understanding ^2^					Engagement ^2^				
	Total (n= 29,473)			1a	2a	3a	4a	5a	1b	2b	3b	4b	5b
Characteristics	N	%	Weighted ^1^ %	(%)									
**Sex**													
Female	15,459	52.5	50.6	17.4	14.6	12.6	18.3	9.1	21.0	17.0	19.2	19.4	16.2
Male	14,014	47.5	49.4	14.7	14.4	13.0	22.2	8.5	15.6	12.0	13.9	17.3	14.3
Missing	0	0.0	0.0										
**Age**													
25-44 years	8,102	27.5	36.6	12.8	14.7	10.2	17.3	8.2	19.6	16.1	18.6	19.2	15.2
45-64 years	12,701	43.1	38.6	15.5	15.5	11.8	20.4	8.5	18.3	14.5	17.2	18.1	15.3
65-84 years	8,046	27.3	22.4	20.7	11.8	17.6	23.4	9.4	15.4	11.1	11.5	16.3	14.6
= > 85 years	624	2.1	2.4	41.9	19.3	32.9	36.9	19.8	24.1	20.6	21.3	29.1	25.7
Missing	0	0.0	0.0										
**Origin**													
Danish	28,400	96.4	93.6	15.6	14.2	11.9	19.7	8.2	17.7	13.9	16.2	17.8	14.6
Immigrant	1,073	3.6	6.4	22.6	19.2	26.1	27.2	17.3	27.8	23.5	23.0	26.5	25.2
Missing	0	0.0	0.0										
**Mother tongue**													
Danish	27,565	93.5	90.3	15.4	14.0	11.8	19.6	8.1	17.4	13.7	16.0	17.7	14.5
Other language	1,441	4.9	8.0	22.1	18.5	24.0	26.6	14.9	26.8	22.0	21.6	24.6	23.3
Missing	467	1.6	1.8										
**Cohabitation**													
Living with others	23,755	80.6	75.9	14.2	13.7	11.4	19.2	8.0	17.1	13.6	16.2	17.3	14.2
Living alone	5,078	17.2	21.9	22.6	17.6	17.9	23.7	11.7	22.6	17.9	18.2	22.1	19.1
Missing	640	2.2	2.2										
**Education level**													
Low	5,507	18.7	18.0	33.9	20.9	28.6	31.9	17.7	25.2	20.9	20.3	26.9	23.4
Medium	14,718	49.9	48.4	14.6	14.7	11.7	20.8	8.0	18.0	13.8	16.2	17.8	14.8
High	8,319	28.2	30.2	7.5	10.3	4.6	12.0	4.4	14.4	11.4	15.1	14.2	10.9
Missing	929	3.2	3.5										
**Income (DKK)** ^**3**^													
0-149,000	6,302	21.4	22.2	24.5	17.7	21.7	26.8	14.2	23.8	19.8	19.3	23.4	21.2
150,000 -374,000	14,417	48.9	48.4	15.6	14.4	11.7	19.4	8.0	18.9	14.4	17.1	18.6	15.0
375,000-699,000	6,132	20.8	20.4	7.6	11.8	5.6	15.5	4.9	11.8	9.3	13.3	13.0	10.3
>700,000	1,054	3.6	3.6	4.6	11.7	3.6	12.8	3.7	10.2	8.0	12.3	9.8	7.9
Missing	1,568	5.3	5.4										
**GP visit last year** ^**4**^													
Yes	22,360	75.9	74.8	17.1	15.5	13.5	20.9	9.1	20.0	15.1	17.0	19.1	15.9
No	6,663	22.6	23.6	12.5	11.5	10.6	17.9	7.7	12.7	12.5	15.3	16.1	13.4
Missing	450	1.5	1.6										

^1^The sample is weighted based on register data to represent the population of the Central Denmark Region 2013.

^2^See Table 1 for description of items and scales.

^3^Personal annual gross income. Exchange rate: 7.46 DKK/1 €.

^4^At least one visit to the general practitioner within the last year.

### Difficulty in undertaking core health literacy tasks

The population-weighted difficulty level of each item is presented in Table 2. The items that were rated least and most difficult were both found in the scale ‘Understanding’; the item with the highest difficulty level was: ‘Read and understand all the information on medication labels’ (4a) 20.2%, 95% CI: 19.6-20.8, and the easiest task was ‘Understand what healthcare providers are asking you to do’ (5a) 8.8%, 95% CI: 8.4-9.2. The items perceived most difficult in the ‘Engagement’ scale were: ‘Make sure that health care providers understand your problems properly’ (1b) 18.3%, 95% CI: 17.7-18.8 and ‘Discuss things with healthcare providers until you understand all you need to’ (4b) 18.3%, 95% CI: 17.8-18.9. The task perceived most easy in the ‘Engagement’ scale was: ‘Feel able to discuss your health concerns with a healthcare provider’ (2b) 14.5%, 95% CI: 14.0-15.0.

### Core health literacy tasks by socio-economic characteristics

**Figure 1:**
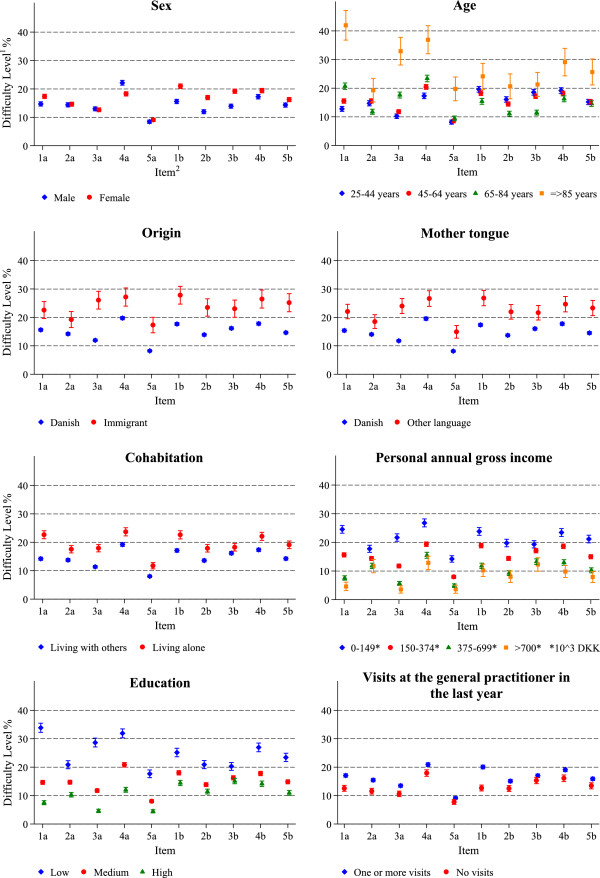
**Population-weighted difficulty level of each item by socio-economic characteristic, Central Denmark Region 2013.** ^1^Difficulty level is calculated as the population-weighted proportion reporting difficult or very difficult. The sample is weighted based on register data to represent the population of the Central Denmark Region 2013. ^2^See Table 1 for description of items and scales.

Table 3 and Figure 1 show population-weighted crude estimates of difficulty levels by socio-economic characteristic for each item. Overall, the difficulty level of the ten items varied between subgroups in the population. Regarding sex, women reported somewhat higher difficulty than men in all items in the ‘Engagement’ scale, especially in items 1b, 2b and 3b that reflect the quality of the communication with healthcare providers. In the ‘Understanding’ scale, the levels were quite similar, except for item 4a, where men perceived more difficulty than women in understanding information on medication labels.

For age, the difficulty level was rather similar across the ages of 25–64 years. The 65–84 year olds perceived more difficulty than the younger age groups regarding the functional skills; filling out medical forms (1a) and reading and understanding health information (3a), but perceived less difficulty than the younger age groups regarding items that reflected a direct contact with healthcare providers (1b-4b), as well as following instruction from health care providers (2a). Individuals aged 85 years or older perceived most items as being more difficult than the younger age groups. Among the oldest individuals, the highest difficulty levels were found for the functional skills reflected in items 1a, 3a, and 4a, where about one third perceived the items as difficult or very difficult. However, also discussing things with health care providers (4b) was rated particularly difficult by the oldest age group.

Both immigrants and the group with non-Danish mother tongue perceived more difficulty than Danes in all items. The largest difference was in reading and understanding health information (3a) where immigrants were more than twice as likely to report difficulties. People who live alone also reported more difficulties across all items than those who live with others. Regarding income and education, there was a clear pattern of an increasing difficulty level with less education or lower income across all items. The most striking pattern was a more than four-fold difference in difficulty level between the lowest and highest education group in the tasks of filling medical forms correctly (1a) and of reading and understanding health information (3a). Those who had visited a general practitioner within the last year perceived all items as slightly more difficult than those, who had not.

### Association between socio-economic factors and two health literacy scales ‘Understanding’ and ‘Engagement’

Table 4 shows results of the linear regression analyses with scale means of the two health literacy dimensions as dependent variables. In Model 1, for each scale, the univariate associations are reported. Model 2 then reports the results when including all the socio-economic factors in the table and the possible confounder ‘visit at the general practitioner in the last year’.

**Table 4:** **Associations between health literacy scales and socio-economic characteristics**

	Understanding ^1^				Engagement ^1^			
	Univariate ^2^		Adjusted ^3^		Univariate ^2^		Adjusted ^3^	
	β	(95% CI)	Β	(95% CI)	Β	(95% CI)	β	(95% CI)
**Sex (reference group: female)**								
Male	-0.01	(-0.03, 0.00)	**-0.04**	(-0.06, -0.03)	**0.06**	(0.04, 0.08)	**0.03**	(0.01, 0.05)
**Age (reference group: age 25–44)**								
45-64	**-0.06**	(-0.08, -0.04)	**-0.03**	(-0.04, -0.01)	0.00	(-0.02, 0.02)	0.02	(-0.00, 0.04)
65-84	**-0.08**	(-0.10, -0.06)	**0.07**	(0.04, 0.09)	**0.06**	(0.04, 0.08)	**0.18**	(0.16, 0.21)
= > 85	**-0.34**	(-0.42, -0.27)	**-0.13**	(-0.22, -0.05)	**-0.14**	(-0.21, -0.06)	0.07	(-0.02, 0.15)
**Origin (reference group: Danish)**								
Immigrant	**-0.14**	(-0.19, -0.09)	**-0.09**	(-0.17, -0.02)	**-0.18**	(-0.22, -0.13)	**-0.09**	(-0.17, -0.01)
**Mother tongue (reference group: Danish)**								
Other language	**-0.12**	(-0.16, -0.08)	-0.02	(-0.08, 0.04)	**-0.15**	(-0.19, -0.11)	-0.03	(-0.10, 0.03)
**Living situation (reference group: With others)**								
Alone	**-0.11**	(-0.13, -0.09)	**-0.05**	(-0.07, -0.03)	**-0.08**	(-0.11, -0.06)	**-0.06**	(-0.09, -0.04)
**Education level (reference group: High)**								
Medium	**-0.21**	(-0.23, -0.20)	**-0.19**	(-0.21, -0.18)	**-0.13**	(-0.15, -0.11)	**-0.13**	(-0.15, -0.11)
Low	**-0.44**	(-0.47, -0.42)	**-0.38**	(-0.41, -0.35)	**-0.26**	(-0.29, -0.24)	**-0.25**	(-0.28, -0.22)
**Income (DKK)** ^**4**^ **(reference group: >700,000)**								
0-149,000	**-0.37**	(-0.42, -0.33)	**-0.25**	(-0.30, -0.21)	**-0.32**	(-0.37, -0.28)	**-0.26**	(-0.31, -0.21)
150,000 -374,000	**-0.26**	(-0.30, -0.22)	**-0.18**	(-0.22, -0.14)	**-0.25**	(-0.29, -0.21)	**-0.18**	(-0.23, -0.14)
375,000-699,000	**-0.13**	(-0.17, -0.09)	**-0.10**	(-0.14, -0.06)	**-0.12**	(-0.16, -0.08)	**-0.09**	(-0.13, -0.05)

Mean scale scores allowing for two missing responses are used as dependent variable in the regression models; statistically significant differences are printed in bold (p< 0.05). The sample is weighted based on register data to represent the population of the Central Denmark Region 2013.

^1^See Table 1 for description of scales.

^2^Unadjusted models of the association between each socio-economic characteristic for the scales ‘Understanding’ and ‘Engagement’. Number of individuals varies for the different models due to missing data in the respective socio-economic factor. ‘Understanding’: n= 26,557-27,512. ‘Engagement’: n= 26,589-27,549.

^3^Model adjusted for all the socio-economic characteristics in table and for the confounder ‘visit at the general practitioner in the last year’. ‘Understanding’: n= 25,486, ‘Engagement’: n= 25,514.

^4^Personal annual gross income. Exchange rate: 7.46 DKK/1 €.

Unadjusted and adjusted models, Central Denmark Region 2013.

The significance of sex varied across scales. For ‘Understanding’, men compared with women had a lower mean level in the adjusted model but for ‘Engagement’, men had a higher mean score than women in the adjusted model.

Regarding age, individuals aged 65–84 years had higher mean scores (i.e. perceived less difficulties) compared with the reference group (25–44 years) in both scales, when adjusting for all the selected covariates. The adjusted results for the age group 45–64 years differed between the scales. For ‘Understanding’, age between 45–64 years was associated with a lower mean level compared with the 25–44 year olds, but for ‘Engagement’, no difference was observed between this age group and the reference group. The same pattern was seen for the oldest age group, with lower mean levels in ‘Understanding’ but no difference in ‘Engagement’ compared with the youngest.

In the unadjusted models both origin and mother tongue were associated with the two health literacy scales, but in the adjusted models including both measures of ethnicity, only origin remained significant, with lower mean levels among immigrants. Also, when adjusting for other factors, individuals who were living alone had lower scores compared with those who live with others.

The largest differences between subgroups were found for income and education level. For both scales, lower income and education level were associated with lower mean scores, when adjusting for other socio-economic factors.

## Discussion

Danish estimates of two key dimensions of health literacy are now available showing that 10-20% of the Danish population perceive difficulties in tasks related to: ‘Ability to understand health information well enough to know what to do’ and ‘Ability to actively engage with healthcare providers’. In people from lower socio-economic groups, those with non-Danish ethnicity, and older people, the proportion reporting difficulties is as high as 20 to 40%. All investigated socio-economic characteristics including sex, age, the two measures of ethnicity, education, income, and cohabitation were independently associated with the two investigated health literacy dimensions.

Given that this study was undertaken in country with a universal healthcare system with multiple policies promoting patient empowerment, patient centred care, user involvement in health planning, and health communication it is concerning that a significant part of the population is reporting difficulties in functional health literacy tasks and in engaging with healthcare providers. Especially considering that those with very poor literacy skills may have been unable to participate due to the self-administered written format. Our results may reflect the increasing complexity of medical treatments, in combination with a healthcare system that constraints resources such as time, staff and money. Furthermore, the OECD Skills Survey from 2012 found that one in six individuals in Denmark has a poor literacy level [33] and Denmark ranged below otherwise comparable Nordic countries as Norway and Sweden [34]. This may add to the explanation to our findings, especially for the functional scale.

The validation study of the HLQ™ [11], undertaken in a healthcare system similar to the Danish with universal access and primary care as entry point, provide data on the percentage of respondents rating items as difficult or very difficult. In the scale ‘Understanding’ the percentage varied between 8–16%, compared to 8–20% in our study, and in the scale ‘Engagement’ it varied between 15–24% compared to 15–24%. In both studies, the most difficult item in the scale ‘Understanding’ was: Read and understand all the information on medication labels (4a) and in the scale ‘Engagement’: Discuss things with healthcare providers until you understand all you need to (4b). However, care should be taken comparing the two studies directly, as we did not use the most extreme response category ‘Cannot do’. In a comparison of eight different European countries, the recent HLS-EU [21] study classified 29% to 62% of the population as having inadequate or limited health literacy depending on the specific country. Other studies from the U.S. and Australia have found that the prevalence of low or suboptimal health literacy, as categorized by the authors, ranged from as low as 7% to a high of 60% [15, 22, 23].

The socio-economic gradient found in our study is clear and previous studies of either self-reported difficulty or abilities measured by functional tests have also documented such gradients [21–23, 35]. In Denmark, 98% of the population have an assigned general practitioner. These follow common rules, medical recommendations, and budgetary agreements. Furthermore, tertiary care is almost exclusively carried out by public hospitals. Therefore, it is likely that the observed social gradient primarily reflects actual differences in individual competencies rather than differences in for example quality of treatment and patient support. It is important to note that the response rate to the survey was 63.6% and non-respondents are more likely to comprise the more socially deprived groups. Nonetheless, the social gradient is clear and future studies would benefit from focused research protocols that include and oversample the ‘hard to reach’ groups.

Previous studies have also found that education is strongly linked to functional health literacy skills and the ability to act upon health information [9, 10], and people with lower education have been found to have lower health literacy in comparison to people with higher education [7, 22, 23]. A recent study suggested that health literacy is a likely mediator for the association between education and health [36]. However, programs have shown that health literacy competencies can be achieved even in individuals with low literacy [10]. Income is the social status indicator that most directly measures material living standards, but is interlinked with other indicators such as employment status, job type, access to health promoting services, self-esteem, and to relative social standing in society [37]. Both education and income being significant in the same model shows that health literacy is tied to more complex social structures than can be described by education alone, which is in line with the complex frameworks for health literacy [9, 38]. Among ethnic minority groups, language can be a barrier to communication with health care providers and make obtaining and processing oral and written health information difficult and thereby lead to low health literacy [39]. We found that, after adjustment for mother tongue, origin remained associated with both ‘Engagement’ and ‘Understanding’. This indicates that other aspects such as perception of health and disease, self-efficacy and other personal resources, or quality of care may determine ethnic differences in health literacy. Living alone was also associated with a lower level of health literacy. Health literacy has been found to be distributed through family and social networks [40], where resources such as knowledge, support for health decision making and communication with health care professionals are passed on between close relations. Thereby, low health literacy may well be compensated by support through family and friends [11].

Older age has often been associated with lower health literacy levels [21, 23], but this study found that compared to the 25–45 year olds, the age group 45–65 years reported less difficulties in ‘Engagement’ and ‘Understanding’. In Denmark all citizens are affiliated with a general practitioner in their geographical region [41]. Therefore an established relationship with their general practitioner, together with longer experience in navigating the health care system, may have strengthened the 45–65 year olds capabilities. For the scale ‘Understanding’, age 85 years or older was significantly associated with lower health literacy levels, but this was not found in the ‘Engagement’ scale. Functional health literacy might decrease with decreasing cognitive abilities of the elderly, and older individuals might face increasingly complex health problems. However, this may not affect their perception of quality of the dialogue with healthcare providers. It is also possible that people in this older generation do not expect to be a participating partner with the health professional and therefore perceive the tasks in the interaction scale as less problematic. Regarding sex, previous studies have found either no differences, or that one of the sexes is more likely to have low health literacy [21, 23, 42]. This study found that the influence of sex was modest and depended on the specific scale. The HLS-EU study found that women compared to men have slightly higher health literacy levels [21].

Our results indicate a need for population level interventions at two levels; optimizing the health system and promoting health literacy in the general population, for example through school-based interventions. The two dimensions of health literacy reflect an individual’s ability to utilize health information actively in decision making about health, and the ability to be proactive and feeling in control in relationships with healthcare providers. Our analysis of specific items guides the improvement of the health system. For example, the perceived difficulty of discussing problems and feeling understood by the health care provider may well reflect a lack of time in the consultation, or lack of communication training of the health care providers. In the functional dimension, our results for example indicate a need for improving the information on medication labels.

### Strength and limitations

This study is the largest health literacy study of individual respondents to date, using a robust measure of health literacy, and employing a sampling frame that was population-based and weighted. The large sample allowed us to perform a nuanced investigation at both the scale and item level, and to investigate the two health literacy dimensions across several socio-economic factors. Furthermore, applying population weights compensated for non-response which made our results representative to the general population. While the cross sectional design of this study limits conclusions about causal associations, it identified vulnerable population groups and provided new insights into plausible individual, healthcare and public health interventions that are required. These population data can be used as norms or benchmarks and can inform future surveys in countries with comparable health systems. There is a need for follow-up studies to develop a better understanding the causal pathway between social status and health literacy as well as for studies that also include analysis of contextual factors. Experimental studies are also needed to identify appropriate interventions.

We used health literacy questions that were developed and translated using a validity driven approach, and they worked well when applied in the population health survey. As opposed to the original HLQ™ the research team together with the HLQ™ authors chose not to include the extreme response category ‘cannot do’. The very low frequency of endorsement of the ‘very difficult’ response option at 1-3% across items (Table 2) indicates that the fifth response option would have been redundant and would not have improved the sensitivity of the survey, nor the conclusion of the results. The category ‘easy’ was used very often. This might impair the possibility to detect differences between groups of people with high health literacy if the questionnaire is used in smaller samples. Therefore, there may be a need to revise response categories in future studies. The self-reported difficulty format worked well in this large survey and gave a nuanced picture of the challenges the public perceive within the two dimensions. It is important to note that the self-report format is not intended to capture actual skills, but instead reflects what the individual experiences in relation to the health literacy demands in their environment, given whatever level of skills they may have. Some studies have shown that tests of functional skills correlate well with self-reported measures [21, 43], but future studies may be strengthened by including the full range of robust psychometric health literacy scales in the HLQ™ alongside functional tests. Nonetheless, in the current study we have provided fine-grained indicators of two dimensions of health literacy that provide specific and important data of immediate relevance for primary care health services planning and priorities.

Even though the study applied population weights, a slight overestimate of the levels of the health literacy dimensions is possible, as those with very low literacy skills or poor Danish language skills may not have been able to participate. In fact, ability and motivation to fill out at health survey can in itself be viewed as a health literacy competence, and applying a self-administered health literacy survey may therefore exclude the most vulnerable groups. Minimal bias due to missing data on socio-economic characteristics is expected, as this was generally low.

The study was limited to two key dimensions of health literacy. It is possible that more striking elements of health literacy strengths and weaknesses may emerge through further investigation of the full range of health literacy concepts present within the HLQ™. It is critical that future studies investigate the relationship between health literacy and health outcomes.

## Conclusions

In Denmark, around 10 to 20% of the general population perceive difficulties in key health literacy dimensions; ability to understand information well enough to know what to do, and ability to actively engage with healthcare providers. Perceived health literacy difficulties are markedly higher in people above 85 years, people with lower income and education level, people who live alone and in people with non-Danish ethnicity. This study provides new and fine-grained information on the health literacy needs across the general population. In particular, the findings reveal a need for population level interventions especially in support of vulnerable and disadvantaged groups and for health policy responses to optimise the health system in Denmark and similar countries.

## Abbreviations

CI: Confidence Intervals
DKK: Danish Kroner
HLQ™: Health Literacy Questionnaire
HLS-EU: European Health Literacy Survey
ISCED: International Standard Classification of Education
SD: Standard Deviation.
